# Correlation between Behavioural and Psychological Symptoms of Alzheimer Type Dementia and Plasma Homocysteine Concentration

**DOI:** 10.1155/2014/383494

**Published:** 2014-06-04

**Authors:** Zhanjie Zheng, Jindong Wang, Lei Yi, Hui Yu, Lingli Kong, Weizhen Cui, Hong Chen, Chunxia Wang

**Affiliations:** ^1^Department of Geriatric, Qingdao Mental Health Center, Qingdao 266034, China; ^2^Office of Director of Hospital, Qingdao Mental Health Center, Qingdao 266034, China

## Abstract

The relationship between plasma homocysteine and behavioral and psychological symptoms of dementia (BPSD) has not been specifically investigated in previous research. In this study, we compared plasma homocysteine (Hcy) among 40 Alzheimer's disease (AD) patients with BPSD, 37 AD patients without BPSD, and 39 healthy controls. Our results evidenced that the plasma homocysteine levels in AD patients with BPSD and without BPSD were higher than healthy controls and that the plasma homocysteine concentration in AD patients with BPSD was the highest among the three groups. Significant correlation between plasma homocysteine concentration and cognitive decline and duration of dementia was observed, but there was no correlation between BPSD and cognitive dysfunction or duration of dementia. In conclusion, this study showed for the first time that BPSD were associated with plasma homocysteine concentration in Alzheimer's dementia, and the results supported that hyperhomocysteine may take part in the pathogenesis of BPSD.

## 1. Introduction

Alzheimer's disease (AD) is characterized by a progressive loss of learning and memory processes and alterations in spatial abilities, confusion, and disorientation. The most known hypotheses about the etiology of AD are that neurodegeneration begins with an abnormal processing of amyloid precursor protein (APP), resulting in production, aggregation, and deposition of the peptide A*β*, thus facilitating the formation of senile plaques and neuronal death [1]. In recent years, most of the researches have great concerns about the cognitive dysfunction of AD patients, while ignoring their behavioral and psychological symptoms of dementia (BPSD), and even the BPSD have been more easily misdiagnosed as a functional mental disorder. Behavioral and psychological symptoms of dementia consist of perception, thought content, mood, and behavior disorders of dementia patients [2, 3]. In 1994, Tariot suggested that mental abnormal or behavioral disorders of dementia, like the cognitive dysfunction, were the core symptoms of dementia [4]. According to the epidemiological survey, 70 to 90 percent of AD patients may experience delusions, hallucinations, agitation, attacks, and other behavioral and psychological symptoms during the course of illness [5].

Most AD patients have BPSD at some stage of their disease [6]. It is the most frequent cause of hospitalization and life quality deterioration for AD patients [7]. Moreover, these symptoms, rather than the memory impairment which could be recognized, cause the most distressing and challenging aspect of the disease and are likely to make the greatest contribution to patients families' burden [8]. In addition, the management of BPSD is very challenging, since there is no licensed drug specifically used for the treatment of these symptoms, and other optional drugs can put patients at risk. Therefore, to confirm whether these symptoms are related to hyperhomocysteinaemia and/or deficiencies in vitamin B12 and folate is of great importance in looking for another efficient medication for AD patients with BPSD.

Decreased serum vitamin B12 and folate concentrations have been reported in Alzheimer's disease patients [9, 10] and psychiatric disorders [11]. The relationships between concentration of serum vitamin B12, folate, and BPSD of dementia were previously assessed by other investigations, but no significant correlation was found [12, 13]. However, no studies have found the correlation between plasma homocysteine concentration and BPSD of dementia patients. Therefore, there is a consistent conclusion that the measurement of homocysteine is essential, especially, since it is speculated that serum vitamin B12 and folate may not reflect the actual status of these vitamins in brain [14].

Homocysteine is an important amino acid connected to vitamin B12 and folate. It is reversibly formed and secreted in the metabolism and can also be used as an effective neurotoxin possibly mediated by increased generation of free radicals or by calcium influx through NMDA receptor channels [15]. Overall, hyperhomocysteine is caused by in vivo genetic or nongenetic metabolic disorder. The toxic effects of hyperhomocysteinaemia have been suggested in cardiovascular and cerebrovascular diseases [16, 17]. In addition, studies have shown that increased plasma homocysteine is associated with the severity of cognitive dysfunction and dementia [18, 19].

Interestingly, hyperhomocysteinaemia has been directly related to psychosis, depression, and other psychiatric disorders [19–23]. There was only one study which reported the correlation between homocysteine and BPSD [24], but we have found no investigations in hyperhomocysteine among AD patients with BPSD in China.

In scope of the recent findings that reported the relation between homocysteine and psychiatric symptoms and potential suggestions that homocysteine measurement may provide a better estimate of tissue activities of vitamin B12 and folate, we compared plasma homocysteine concentration in AD patients with and without BPSD and healthy participants to explore the possible different concentrations of homocysteine in AD patients with BPSD. Moreover, we investigated whether a specific correlation exists between homocysteine concentration and clinical symptoms.

## 2. Material and Methods

### 2.1. Participants

The sample consisted of 116 participants: 40 Alzheimer's disease (AD) patients with BPSD, 37 AD patients without BPSD, and 39 healthy controls. Between May 2010 and March 2012, AD patients were all recruited from the outpatients and inpatients of Qingdao Mental Health Center. All subjects met the criteria for AD in baseline according to the fourth edition of the Diagnostic and Statistical Manual of Mental Disorders (DSM-IV). And the score of Mini-Mental State Examination (MMSE) should be no more than 24. Exclusion criteria included patients older than 80 years; patients receiving vitamin B12 injections or folate tables; patients with other forms of dementia such as vascular, frontotemporal, and Lewy body; patients receiving methotrexate and/or anticonvulsants including carbamazepine, phenytoin, phenobarbitone, or valproate; patients with moderate to severe renal failure; patients with thyroid disease; and patients on special diets. The eligible patients were initially divided into two study groups based on the presence or absence of BPSD. In the BPSD group, each patient had at least one symptom of perception, thought content, behavior, or mood disorders. These behavioral and psychological symptoms of all AD patients were judged by the professional psychiatrists.

Healthy controls were the healthy elderly with routine health examination in the corresponding period. Inclusion criteria were absence of alcohol and substance abuse, no relevant neurological disease, MMSE scores above 27 of 30, and without any personal and family history (first-degree relatives) of psychiatric DSM-IV Axis I disorders (confirmed with the Mini-International Neuropsychiatric Interview (M.I.N.I)).

The study was approved by the local ethics committee and carried out in accordance with the Declaration of Helsinki (current version: Somerset West, 1996).

### 2.2. Biochemical Investigation

Venous blood (2 mL) was taken from all participants in the morning after an overnight fast. Blood samples were collected into tubes without anticoagulant and allowed to clot at room temperature for 1 hour. Serum was separated by centrifuge and then stored in the refrigerator at −30°C. All the samples were numbered, and plasma homocysteine levels were measured by the specific person of our hospital's laboratory. Homocysteine concentrations were assayed by enzymatic cycling assay (DiaSys Diagnostic Systems GmbH, Germany). The clinical reference range of homocysteine is from 5 to 15 *μ*mol/L, and hyperhomocysteine is defined as homocysteine being more than 15 *μ*mol/L.

### 2.3. Cognitive Assessment

The severity of cognitive impairment was assessed by Mini-Mental State Examination scores (MMSE). The rating scale of MMSE has 11 questions with a total score of 30 points. The cut-off value which could distinguish dementia patients from healthy people was illiteracy—less than 17 points, primary—less than 20 points, more than high school—less than 24 points.

### 2.4. Statistical Analysis

SPSS 18.0 was used for all data entry and subsequent analyses. The analyses included *t*-test, chi-squared test, analysis of variance (ANOVA) with post hoc LSD analysis, and Pearson's linear correlation coefficient when appropriate. The level of statistical significance was set at *P* < 0.05.

## 3. Results

### 3.1. General Information

Demographic and clinical characteristics of patients and controls are shown in Table 1. There was no significant difference in age, gender, and education distributions among the groups. In regard to clinical and cognitive aspects, both “BPSD” and “No BPSD” groups did not differ significantly in illness duration of dementia. Significant differences were observed in MMSE scores among the three groups using one-way analyses of variance (ANOVA). Moreover, with a post hoc analysis, we found that MMSE scores were significantly lower in “BPSD” and “No BPSD” groups than in health controls (*P* < 0.001) and had no difference between the “BPSD” and “No BPSD” groups (*P* = 0.864). Among all subjects taking part in the study there was no significant correlation between age and MMSE score and between illness duration of dementia and plasma homocysteine (*P* > 0.05).

### 3.2. Comparison of Homocysteine Levels

There was a significant difference among the three groups in homocysteine concentration (Table 2). Further post hoc analyses illustrated significant increases in plasma homocysteine concentrations in BPSD and No BPSD groups compared with controls (*P* < 0.001), with significantly higher level in BPSD group than that in No BPSD group (*P* < 0.001). In addition, all 40 BPSD patients were hyperhomocysteine, while only 27 out of 37 patients without BPSD were hyperhomocysteine (73.0%). In contrast, none of the healthy control group was hyperhomocysteine. There were significant differences in frequency ratio of hyperhomocysteine and nonhyperhomocysteine individuals between three groups (*P* < 0.001).

### 3.3. The Relation between Homocysteine and Cognitive Dysfunction

**Figure 1:**
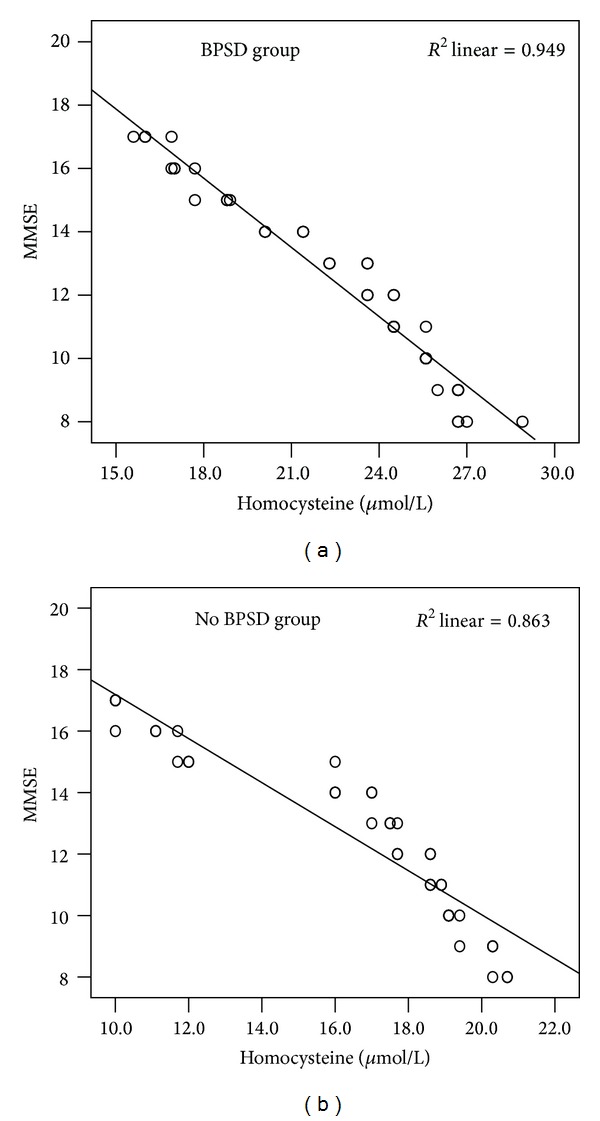
The correlations between MMSE and homocysteine in AD patients. (a) A significant negative correlation between MMSE and homocysteine in BPSD patients group (*r* = 0.974; *P* < 0.001). (b) A significant negative correlation between MMSE and homocysteine in No BPSD patients group (*r* = 0.929; *P* < 0.001). Correlation lines are also shown.

The 40 patients of BPSD group had a mean MMSE score of 12.50 ± 2.91, ranging from 8 to 17 points. The mean MMSE score of the No BPSD group was 12.59 ± 2.77, ranging from 8 to 17. MMSE scores between the two patient groups showed no significant difference (*P* = 0.864). There was a prominent negative correlation between the homocysteine and MMSE score in the BPSD group (*r* = 0.974; *P* < 0.001). We also found a similar significant negative correlation in the No BPSD patients (*r* = 0.929; *P* < 0.001) (Figure 1(a)). As we expected, the correlation between homocysteine and MMSE did not exist in control group (*r* = 0.033; *P* = 0.841) (Figure 1(b)).

### 3.4. The Relation between Homocysteine and Duration of Illness

**Figure 2:**
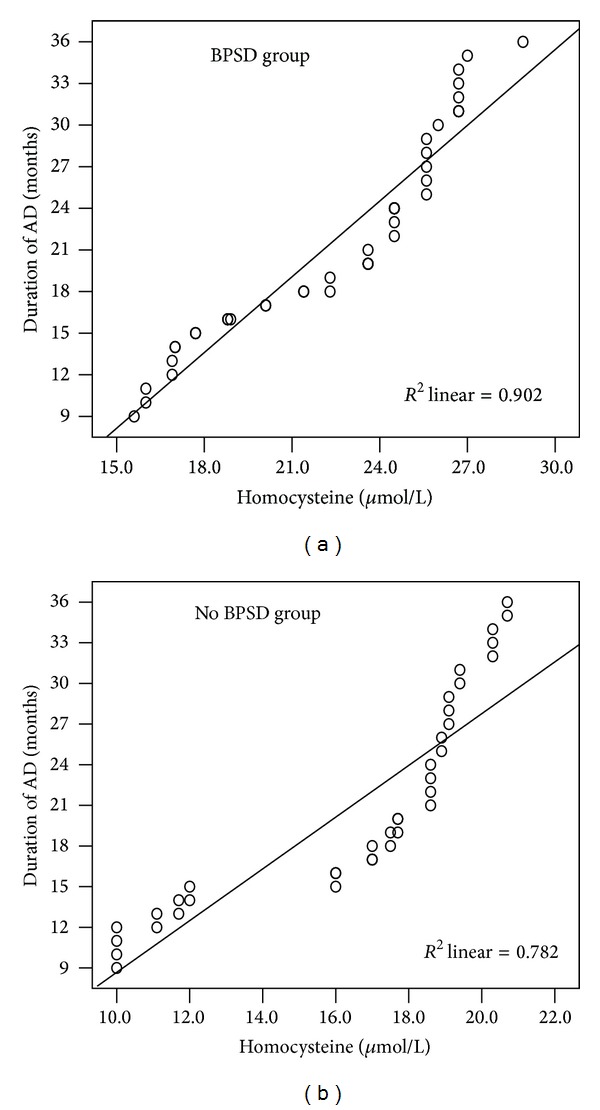
The correlations between duration of AD and homocysteine in AD patients. (a) A significant positive correlation between duration of AD and homocysteine in BPSD patients group (*r* = 0.988; *P* < 0.001). (b) A significant positive correlation between duration of AD and homocysteine in No BPSD patients group (*r* = 0.974; *P* < 0.001). Correlation lines are also shown.

As illustrated in Table 1, there was no significant difference in the duration of AD between the AD patients with and without BPSD. However, we found a significant positive correlation between the homocysteine and the illness duration of AD in the two patient groups (*r* = 0.988; *P* < 0.001; *r* = 0.974; *P* < 0.001, resp.) (Figure 2).

## 4. Discussion

The first important aspect of our finding is that we observed that both “BPSD” and “No BPSD” groups had higher homocysteine concentration than that of control group, while BPSD patients showed higher homocysteine concentration than No BPSD patients. Hence, this study implicated that the concentration of hyperhomocysteinaemia played an important role in pathogenesis of AD. Further, the result suggested that higher level of plasma homocysteine might be related to presence of BPSD in AD patients. Previous study found no significant difference of the homocysteine concentration between BPSD and No BPSD patients [24]. There were very few studies that reported the association between homocysteine and BPSD. We insisted that this was the first study demonstrating that homocysteine was correlated with BPSD. However, there was no consistent conclusion of whether hyperhomocysteine had some relevance with the absence of BPSD in AD patients. In future, more studies are needed to assess the characteristics of the association between homocysteine and BPSD.

The study also found that MMSE scores of the two AD groups were significantly lower than control group, but there was no significant difference between the two patient groups. In the AD patient groups, there was a highly significant negative correlation between the homocysteine and MMSE. However, Tabet's study [24] failed to find this correlation. The mean MMSE score was significantly lower in the BPSD group than that of No BPSD group but showed no significant correlation between MMSE score and homocysteine. MMSE score could reflect the degree of cognitive decline in patients. With the decline in MMSE scores, the degree of cognitive impairment in patients was aggravated. The result may indicate that increase of plasma homocysteine can lead to aggravation of the cognitive dysfunction in AD patients, which is consistent with previous reports [25]. However, there was no correlation between MMSE score and BPSD. All our findings supported that homocysteine is associated with cognitive decline [26–28].

In our study, the range of the MMSE score was from 8 to 17 in the AD patients, which was obviously lower than results of a previous report [24]. In that report, the MMSE score ranged from 10 to 24, with higher than 20 scores among sixty percent of the AD participants. There might be some reasons to explain this. MMSE was more vulnerable compared with education level. The years of education in our subjects were all less than six years, while the Tabet study did not provide the education years. The education cultural differences between Eastern and Western countries should also be taken into account. Therefore, the difference of MMSE between two studies cannot rule out the effect of education factor. In addition, the requirement of life quality may also have difference, which meant that the inconsistency of the study results might be caused by the severity of the AD disease when the patients seek medical treatment.

Moreover, plasma homocysteine concentration was positively correlated with the illness duration of AD patients. It showed that homocysteine gradually increased with the progression of AD, which was consistent with previous findings [29]. As the etiology of AD is complex, AD is caused by many factors working together. Homocysteine may be just one of the factors and not the main or primary one. However, it can contribute to the occurrence of AD or lead to the aggravation of a variety of pathophysiological damage. Finally, all these would make the disease more severe.

While our study has found some valuable results, there are still some limitations in our study. First is that the assessment of patients with and without BPSD was based only on the subjective judgment of psychiatrist. Although the study fixed the same psychiatrist to assess all patients to reduce the errors between raters as much as possible, we did not use an objective investigation scale in the assessment of BPSD. A further limitation was that we did not have the concurrent measurement of the serum vitamin B12 and folate. Previous studies have demonstrated no link between vitamin B12 and folate with BPSD [12–14], but the simultaneous determination of vitamin B12 and folate can make the results of this study exclude interference and be more convincing.

In the study, we had taken some efforts to ensure that the plasma values of homocysteine were obtained to reflect the accurate reflection of in vivo status. Blood samples of recruited patients were collected in the morning after an overnight fast, which increased the chance of obtaining a “true” fasting blood. Then, these blood samples were transported immediately to the analyzing laboratory in line with the strict protocol set. In addition, none of the patients had any disease that could interfere with food intake or had a disorder affecting food absorption or received special diets. This is in agreement with some reports adopting a strict standard to obtain the most accurate homocysteine concentration [24]. A merit of this study is that we recruited a healthy control group to compare the homocysteine concentration with AD patients. Hence, we were more confident with difference of homocysteine levels among the three groups and excluded the impact of metabolism in human body. Even, some studies recognized that the absence of a control group to compare the mean homocysteine levels compared to those with AD as a limitation [24].

This study found that the presence of BPSD in AD patients may associate with homocysteine level, but there was no significant correlation between the BPSD and the cognitive dysfunction or duration of illness, which may be caused by the smaller sample size and lack of the efficient assessment tool for AD patients with and without BPSD. In future, there will be other researches which expand the sample size, and a longitudinal study would be conducted to investigate the related factors of BPSD in AD patients. In addition, the simultaneous determination of vitamin B12 and folate is equally important. Establishing whether a specific link exists between hyperhomocysteinaemia and psychiatric symptoms in AD patients may have significant therapeutic potential in discovering the relationship between vitamin B12 and folate supplementation to provide necessary assistance for the prevention and treatment of AD patients with BPSD. Therefore, future research can combine with molecular genetics, candidate genes including MTHFR, CBS, and serine hydroxymethyl transferase (SHMT) together, and integrate the neuropsychological tests with neuroimaging analyses so as to comprehensively reveal the role of high levels of homocysteine in the pathogenesis of BPSD in AD patients.

## Figures and Tables

**Table 1:** Mean (M) and standard deviations (SD) of several demographic and clinical features of the patient and control groups.

	BPSD	No BPSD	Control	*P* value
Sample size (*N*)	40	37	39	
Gender (M/F)	19/21	19/18	19/20	
Age (years)	72.10 ± 4.84	71.78 ± 4.85	71.05 ± 4.69	0.611
Education (years)	2.30 ± 1.31	2.38 ± 1.42	2.33 ± 1.38	0.969
Duration of dementia (months)	21.58 ± 7.45	20.92 ± 7.77		0.706
MMSE	12.50 ± 2.91*	12.59 ± 2.77*	23.46 ± 1.12	0.000*

*Significant difference (*P* < 0.05).

**Table 2:** Fasting plasma homocysteine in the three groups.

	BPSD	No BPSD	Control	*P* value
Numbers (*N*)	40	37	39	
Homocysteine (*μ*mol/L)	22.38 ± 3.89	16.41 ± 3.60	7.98 ± 1.71	0.000*

*Significant difference (*P* < 0.05).

## References

[1] Fuentealba RA, Farias G, Scheu J, Bronfman M, Marzolo MP, Inestrosa NC (2004). Signal transduction during amyloid-*β*-peptide neurotoxicity: role in Alzheimer disease. *Brain Research Reviews*.

[2] Association IP (1996). Research methodological issue in evaluating behavioral disorders of dementia. *International Psychogeriatrics*.

[3] Qu HF, Zhang H, Sheng JH, Gao ZX (2003). Comparison of the behavioral disorder in Alzheimer's disease and vascular dementia patients. *Journal of Clinical Psychological Medicine*.

[4] Tariot P, Blazina L (1994). The psychopathology of dementia. *Neurological Disease and Therapy*.

[5] Wei J, Li HW (2008). Progress in clinical research of Alzheimer's disease. *Journal of Qiqihar Medical College*.

[6] Piccininni M, di Carlo A, Baldereschi M, Zaccara G, Inzitari D (2005). Behavioral and psychological symptoms in Alzheimer’s disease: frequency and relationship with duration and severity of the disease. *Dementia and Geriatric Cognitive Disorders*.

[7] Steinberg M, Sheppard J-M, Tschanz JT (2003). The incidence of mental and behavioral disturbances in dementia: the Cache County Study. *The Journal of Neuropsychiatry and Clinical Neurosciences*.

[8] Rymer S, Salloway S, Norton L, Malloy P, Correia S, Monast D (2002). Impaired awareness, behavior disturbance, and caregiver burden in Alzheimer disease. *Alzheimer Disease & Associated Disorders*.

[9] Quadri P, Fragiacomo C, Pezzati R (2004). Homocysteine, folate, and vitamin B-12 in mild cognitive impairment, Alzheimer disease, and vascular dementia. *The American Journal of Clinical Nutrition*.

[10] Stuerenburg HJ, Mueller-Thomsen T, Methner A (2004). Vitamin B_12_ plasma concentrations in Alzheimier disease. *Neuroendocrinology Letters*.

[11] Bottiglieri T (1996). Folate, vitamin B_12_, and neuropsychiatric disorders. *Nutrition Reviews*.

[12] Engelborghs S, Vloeberghs E, Maertens K (2004). Correlations between cognitive, behavioural and psychological findings and levels of vitamin B_12_ and folate in patients with dementia. *International Journal of Geriatric Psychiatry*.

[13] Whyte EM, Mulsant BH, Butters MA (2002). Cognitive and behavioral correlates of low vitamin B_12_ levels in elderly patients with progressive dementia. *The American Journal of Geriatric Psychiatry*.

[14] Nilsson K, Gustafson L, Hultberg B (1999). Plasma homocysteine is a sensitive marker for tissue deficiency of both cobalamines and folates in a psychogeriatric population. *Dementia and Geriatric Cognitive Disorders*.

[15] Ho PI, Ortiz D, Rogers E, Shea TB (2002). Multiple aspects of homocysteine neurotoxicity: glutamate excitotoxicity, kinase hyperactivation and DNA damage. *Journal of Neuroscience Research*.

[16] Fácila L, Nuñez JE, Bertomeu G. V (2005). Early determination of homocysteine levels in acute coronary syndromes, is it an independent prognostic factor?. *International Journal of Cardiology*.

[17] Virtanen JK, Voutilainen S, Happonen P (2005). Serum homocysteine, folate and risk of stroke: Kuopio Ischaemic Heart Disease Risk Factor (KIHD) Study. *European Journal of Cardiovascular Prevention and Rehabilitation*.

[18] Adunsky A, Arinzon Z, Fidelman Z, Krasniansky I, Arad M, Gepstein R (2005). Plasma homocysteine levels and cognitive status in long-term stay geriatric patients: a cross-sectional study. *Archives of Gerontology and Geriatrics*.

[19] Reif A, Pfuhlmann B, Lesch K-P (2005). Homocysteinemia as well as methylenetetrahydrofolate reductase polymorphism are associated with affective psychoses. *Progress in Neuro-Psychopharmacology and Biological Psychiatry*.

[20] Qian ZK, Jiang CX, Du XD (2009). Relationship between plasma homocysteine, serum folate levels and first-episode schizophrenic patients. *Suzhou University Journal of Medical Science*.

[21] Chen C-S, Tsai J-C, Tsang H-Y (2005). Homocysteine levels, MTHFR C677T genotype, and MRI hyperintensities in late-onset major depressive disorder. *The American Journal of Geriatric Psychiatry*.

[22] Atmaca M, Tezcan E, Kuloglu M, Kirtas O, Ustundag B (2005). Serum folate and homocysteine levels in patients with obsessive-compulsive disorder. *Psychiatry and Clinical Neurosciences*.

[23] Regland B, Abrahamsson L, Blennow K, Grenfeldt B, Gottfries C-G (2004). CSF-methionine is elevated in psychotic patients. *Journal of Neural Transmission*.

[24] Tabet N, Rafi H, Weaving G, Lyons B, Iversen S (2006). Behavioural and psychological symptoms of Alzheimer type dementia are not correlated with plasma homocysteine concentration. *Dementia and Geriatric Cognitive Disorders*.

[25] McCaddon A, Hudson P, Davies G, Hughes A, Williams JH, Wilkinson C (2001). Homocysteine and cognitive decline in healthy elderly. *Dementia and Geriatric Cognitive Disorders*.

[26] Schafer JH, Glass TA, Bolla KI, Mintz M, Jedlicka AE, Schwartz BS (2005). Homocysteine and cognitive function in a population-based study of older adults. *Journal of the American Geriatrics Society*.

[27] Budge MM, de Jager C, Hogervorst E, Smith AD (2002). Total plasma homocysteine, age, systolic blood pressure, and cognitive performance in older people. *Journal of the American Geriatrics Society*.

[28] Prins N, den Heijer T, Hofman A (2002). Homocysteine and cognitive function in the elderly: the Rotterdam Scan Study. *Neurology*.

[29] Nilsson K, Gustafson L, Hultberg B (2002). Relation between plasma homocysteine and Alzheimer’s disease. *Dementia and Geriatric Cognitive Disorders*.

